# MEK1/2 activity modulates TREM2 cell surface recruitment

**DOI:** 10.1074/jbc.RA120.014352

**Published:** 2020-12-25

**Authors:** Jason Schapansky, Yelena Y. Grinberg, David M. Osiecki, Emily A. Freeman, Stephen G. Walker, Eric Karran, Sujatha M. Gopalakrishnan, Robert V. Talanian

**Affiliations:** 1 AbbVie Inc, Cambridge Research Center, Cambridge, Massachusetts, USA; 2 AbbVie Inc, Drug Discovery Science and Technology, North Chicago, Illinois, USA

**Keywords:** Alzheimer’s disease, neurodegeneration, neuroinflammation, microglia, phagocytosis, TREM2, MEK, AD, Alzheimer’s disease, DAM, disease-associated microglia, NHD, Nasu–Hakola disease, KO, knockout, TREM2, triggering receptor expressed on myeloid cells-2

## Abstract

Rare sequence variants in the microglial cell surface receptor TREM2 have been shown to increase the risk for Alzheimer’s disease (AD). Disease-linked TREM2 mutations seem to confer a partial loss of function, and increasing TREM2 cell surface expression and thereby its function(s) might have therapeutic benefit in AD. However, druggable targets that could modulate microglial TREM2 surface expression are not known. To identify such targets, we conducted a screen of small molecule compounds with known pharmacology using human myeloid cells, searching for those that enhance TREM2 protein at the cell surface. Inhibitors of the kinases MEK1/2 displayed the strongest and most consistent increases in cell surface TREM2 protein, identifying a previously unreported pathway for TREM2 regulation. Unexpectedly, inhibitors of the downstream effector ERK kinases did not have the same effect, suggesting that noncanonical MEK signaling regulates TREM2 trafficking. In addition, siRNA knockdown experiments confirmed that decreased MEK1 and MEK2 were required for this recruitment. In iPSC-derived microglia, MEK inhibition increased cell surface TREM2 only modestly, so various cytokines were used to alter iPSC microglia phenotype, making cells more sensitive to MEK inhibitor-induced TREM2 recruitment. Of those tested, only IFN-gamma priming prior to MEK inhibitor treatment resulted in greater TREM2 recruitment. These data identify the first known mechanisms for increasing surface TREM2 protein and TREM2-regulated function in human myeloid cells and are the first to show a role for MEK1/MEK2 signaling in TREM2 activity.

Interest in microglial biology in neurodegenerative disease has intensified since genome-wide association studies revealed multiple microglia-specific gene risk variants for Alzheimer’s disease (AD). Of those, triggering receptor expressed on myeloid cells-2 (TREM2) has emerged as one of the strongest known genetic AD risk factors (1, 2, 3). TREM2 is a myeloid cell surface receptor for which homozygous mutations that eliminate protein expression and/or function result in Nasu–Hakola disease (NHD), an inherited disorder that results in polycystic bone lesions, presenile dementia, and death by the early 50s (4, 5). Heterozygous mutations that may only partially impair TREM2 function, such as R47H, significantly increase the odds ratio for developing AD to about 2.0 to 4.5, similar to that of apolipoprotein E ε4 (1, 2). NHD develops from a complete loss of TREM2 signaling, and while the partial loss-of-function mutations associated with AD do bring forward the age of onset, patients nevertheless are in their late 60s, suggesting a more insidious biological effect. Understanding the function and regulation of TREM2 in microglia could provide insight into AD pathogenesis and may reveal opportunities for therapeutic intervention.

Compromising TREM2 activity in mouse models of AD alters progression and pathology, as well as general microglial health (6, 7, 8). Microglia closely associated with amyloid plaques, also known as disease-associated microglia (DAM), have higher TREM2 expression than homeostatic microglia (9). Development of these DAMs is deficient in TREM2 knockout (KO) mice (10). In AD transgenic mice that are also TREM2 KO, there are fewer microglia associated with plaques (7, 11), a phenomenon also observed in AD patients with mutant TREM2 variants (12). These reduced microglia numbers were associated with a more diffuse plaque morphology, as well as increased neuronal dystrophy (7, 11). Increased amyloid or tau tangle deposition is also frequently observed in both TREM2 KO and R47H AD model animals (13, 14, 15, 16, 17). Functional studies with microglia *in vitro* show that phagocytic capacity toward various substrates, including *E. coli* and fibrillar amyloid beta, is impaired with both TREM2 mutations and KO (18, 19, 20, 21, 22, 23). Migration in both TREM2-deficient and TREM2 R47H microglia is reduced (24, 25, 26), while proinflammatory cytokine expression, basally and upon DAMP/PAMP activation, is upregulated (25, 27). Overall defects in TREM2 signaling can reduce mTOR activation and cellular metabolism (18). TREM2 KO decreases expression of genes associated with lipid metabolism (10, 28), and in cuprizone-induced demyelination results in an accumulation of lipid byproducts (29, 30), likely due to a combination of reduced phagocytosis and poor lipid processing. The broad-ranging impact of TREM2 dysfunction on both general microglial health and risk of AD reinforces the relevance of proper receptor function to preventing AD.

Assessing the biological impact of increased TREM2 signaling is challenging, as there are few strategies available to increase TREM2 activation directly. The lack of bona-fide ligand and/or the promiscuity of the receptor (31) adds uncertainty. Various lipids and lipoproteins have been suggested as substrates (7, 32, 33, 34), but the evidence is mainly from binding rather than functional studies. Increasing TREM2 signaling by alternate means has provided some insight. Ectopic TREM2 expression ameliorates AD symptoms in animal models by reducing plaque deposition (35) and neuroinflammation (35, 36). TREM2 overexpression can also partially alter the transcriptomic profile in DAMs to improve microglial function and reduce AD-associated behavioral deficits in an AD model (37). TREM2 activating antibodies can enhance microglial survival, chemotaxis, and phagocytosis (25, 38). Increasing the half-life of surface TREM2 with an anchoring antibody modulates microglia function and facilitates plaque removal in an AD model (39). Crucially, both TREM2 overexpression and increased receptor surface retention appear well tolerated in animal studies. However, from the perspective of developing therapeutics, both gene therapy and immunotherapies are challenging for CNS applications, and little is known regarding cellular mechanisms that can be used to increase TREM2 in human cells. Alternate approaches to enhance TREM2 signaling are required.

Clinical trials for new AD drugs have so far proven disappointing, driving a major need for therapeutic targets of novel mechanisms. Increasing TREM2 activity in neuroinflammatory diseases, either directly or indirectly, is one such possibility. Indirect methods, such as raising TREM2 cell surface expression to increase TREM2 function, could be an effective alternative to immunotherapies with poor CNS penetrance, or as part of a combination therapy with other TREM2 stimulators. We therefore performed a small-molecule phenotypic screen to identify mechanisms of modulation of cell surface TREM2 in human myeloid cells. We report that MEK1/2 is a unique negative regulator of surface TREM2, possibly acting *via* a noncanonical signaling pathway.

## Results

**Figure 1:**
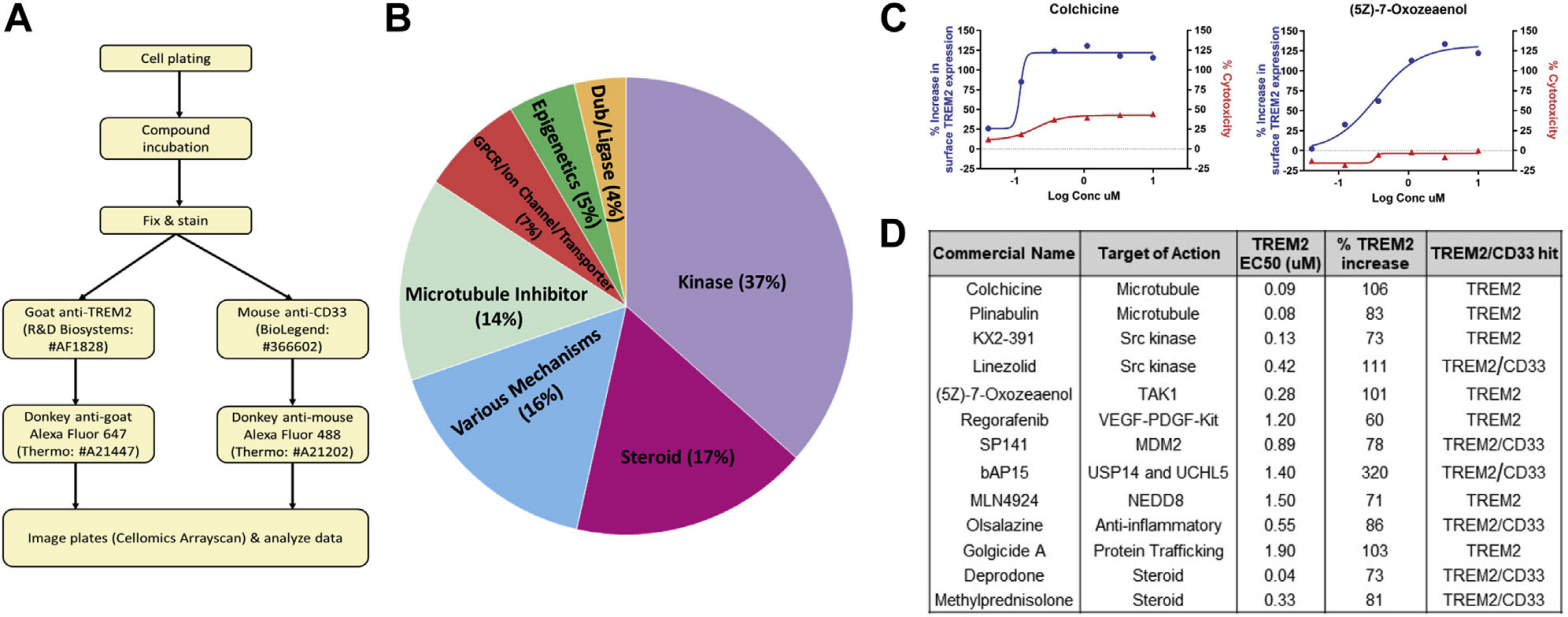
**A small molecule annotated screen finds regulators of TREM2 surface expression.***A*, workflow for TREM2/CD33 screen. *B*, pie chart demonstrating hits based on compound mechanism of action. *C*, example graphs of top TREM2 hits following 6-point compound validation. *D*, tabulation of representative hits.

To find mediators of cell surface TREM2 recruitment, we screened an annotated small-molecule library using THP1-1 cells, a myeloid cell line commonly used as a model for macrophages. The assay workflow is shown in Figure 1*A*. We used a 24 h compound treatment to identify targets and associated signaling pathways that may give a sustained and stable increase in TREM2 expression, as opposed to a quick but potentially transient effect with shorter time points. PMA-differentiated THP1 cells were treated with a 29K annotated compound library composed of FDA-approved compounds, preclinical and clinical candidates, internal bioactive compounds with potent and specific activities, reference compounds for various targets and pathways, and commercially available targeted compound collections. Levels of surface TREM2 were measured *via* a high-content immunofluorescence readout. Cytotoxicity and cell surface CD33 expression was also measured for each compound. CD33 was selected as a general specificity control for TREM2, but also because its functional effects have been suggested to oppose those of TREM2 (28, 40). We found 217 compounds that increased cell surface TREM2 by at least 50%. The hits could be placed into six main categories plus “various mechanisms” (Fig. 1*B*). Inhibition curves for representative compounds show dose-dependent TREM2 increases (Fig. 1*C*), without any associated toxicity. Figure 1*D* tabulates examples of commercial compounds representative of the hits. Many compounds increased both TREM2 and CD33. Since these likely represent mechanisms that stimulate cell surface translocation of proteins generally, and by virtue of the opposing effects of TREM2 and CD33 on phagocytosis, these compounds were not prioritized for further analysis.

**Figure 2:**
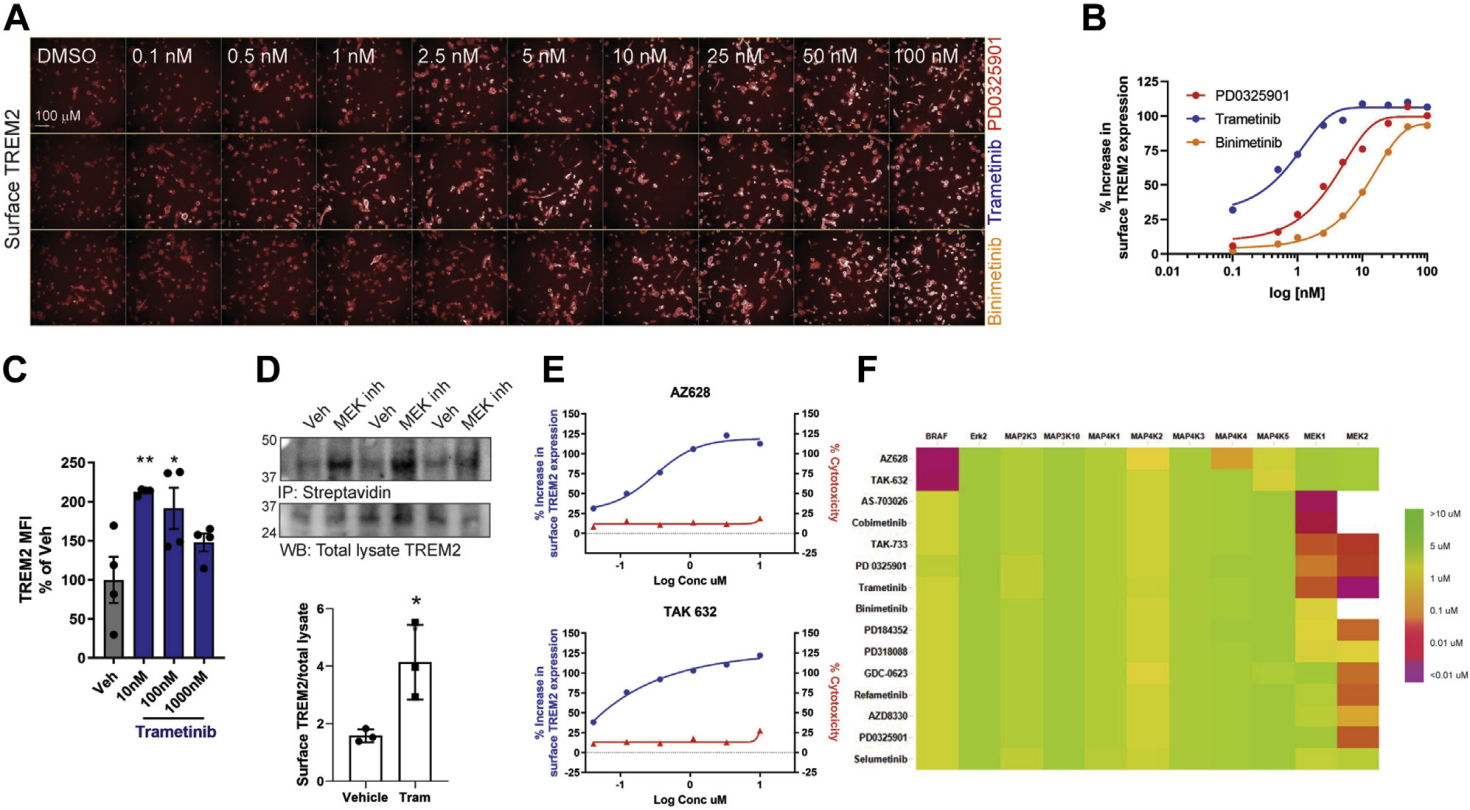
**MEK inhibitors, but not ERK inhibitors, are strong inducers of increased surface TREM2 expression.***A*, images showing levels of surface TREM2 expression following 10-point titrations of MEK inhibitors, starting at 0.1 nM. *B*, representative graph reflects surface TREM2 expression over concentration range for each inhibitor. *C*, surface TREM2 immunostaining detection of THP1 cells treated with MEK inhibitor trametinib, as detected by flow cytometry. *D*, biotinylated cell surface TREM2 was immunoprecipitated with neuravidin following trametinib treatment and surface biotinylation. Levels of surface TREM2 were normalized to total lysate TREM2 as detected by immunoblot (n = 3, ∗*p* < 0.05). *E*, graphs demonstrated 6-point titration using two commercial inhibitors of the protein Raf, a kinase upstream of MEK1/2 kinase. *F*, kinase selectivity profile, using cell-free TR-FRET analysis of select MEK or RAF inhibitors (displayed on side of heatmap). Members of MAPK signaling are displayed along the top of heatmap. Values reflect K_i_ of inhibitors for each kinase, in μM.

The most robust effects were seen with multiple inhibitors of the MEK1/2 kinases. A significant increase in TREM2 was observed at concentrations as low as 10 nM (Fig. 2, *A*–*B*), similar to the IC_50_ values of these compounds for MEK1 enzyme (41). Validation of these compounds by an orthogonal methodology, flow cytometry, involving treating THP1 cells with MEK1/2 inhibitor trametinib at the doses indicated followed by immunostaining, revealed a similar effect (Fig. 2*C*). Surface biotinylation and immunoprecipitation revealed high levels of high-molecular-weight TREM2, reflecting glycosylated surface TREM2, in trametinib-treated THP1 cells compared with vehicle alone (Fig. 2*D*). Analysis of all MEK pathway-related commercial inhibitors in the library revealed multiple MEK inhibitors that significantly increase surface TREM2 expression, including two inhibitors of the upstream Raf enzyme (Table 1, Fig. 2*E*). Kinome inhibition profiling of MEK pathway library compounds suggests that these inhibitors are highly specific for MEK1/2 or Raf (Fig. 2*F*); thus, the observed effect was likely due to on-target inhibition. However, the majority of inhibitors of ERK kinase, the immediate substrate of both MEK 1 and MEK2, did not robustly increase surface TREM2 at any concentration tested (Table 1). Only one ERK inhibitor increased surface TREM2, with an EC_50_ of 1 μM (Table 1); however, this compound inhibits ERK2 enzyme with an IC_50_ value <10 nM (data not shown), suggesting that the observed effect on TREM2 in THP1 cells is most likely off-target. These results suggest that the increased cell surface TREM2 induced by MEK inhibition is through mechanism(s) other than through their predominant substrates, the ERK kinases (42).

**Table 1:** List of compounds in MEK signaling pathway used in small-molecule screen

MEK1/2 and upstream			Downstream of MEK1/2			
Inhibitor	TREM2 EC50 (μM)	Target	Inhibitor	TREM2 EC50 (μM)	Target	% TREM2 increase
PD 0235901	<0.1	MEK	TCS ERK 11e	1	ERK	97.3
Trametinib	<0.1	MEK	SCH772984.0	10	ERK	31.8
AS-703026	<0.1	MEK	LY-3214996	10	ERK	16.4
Cobimetinib	<0.1	MEK	AX 15836	10	ERK	15.1
RO5126766	<0.1	MEK	XMD 8–92	10	ERK	15
PD318088	<0.1	MEK	ERK-IN-1	10	ERK	14.6
AZD8330	<0.1	MEK	BIX 02180	10	ERK	11.7
TAK-733	<0.1	MEK	FR 180204	10	ERK	10.9
GDC-0623	<0.1	MEK	DEL 22379	10	ERK	7.9
BI-847325	<0.1	MEK				
TAK-632	0.11	Raf				
Binimetinib	0.21	MEK				
AZ628	0.32	Raf				
PD184352	0.33	MEK				
PD184352	0.33	MEK				
Refametinib	0.36	MEK				
Selumetinib	0.45	MEK				

Several MEK1/2 or upstream RAF inhibitors had maximal effect on surface TREM2 expression at low or modest nanomolar concentrations. All upstream inhibitors listed increased surface TREM2 by ≥100%, while most inhibitors of ERK, the kinase immediately phosphorylated by MEK1/2, had no effect. Amount of actual TREM2 increase was listed for ERK inhibitors for context.

**Figure 3:**
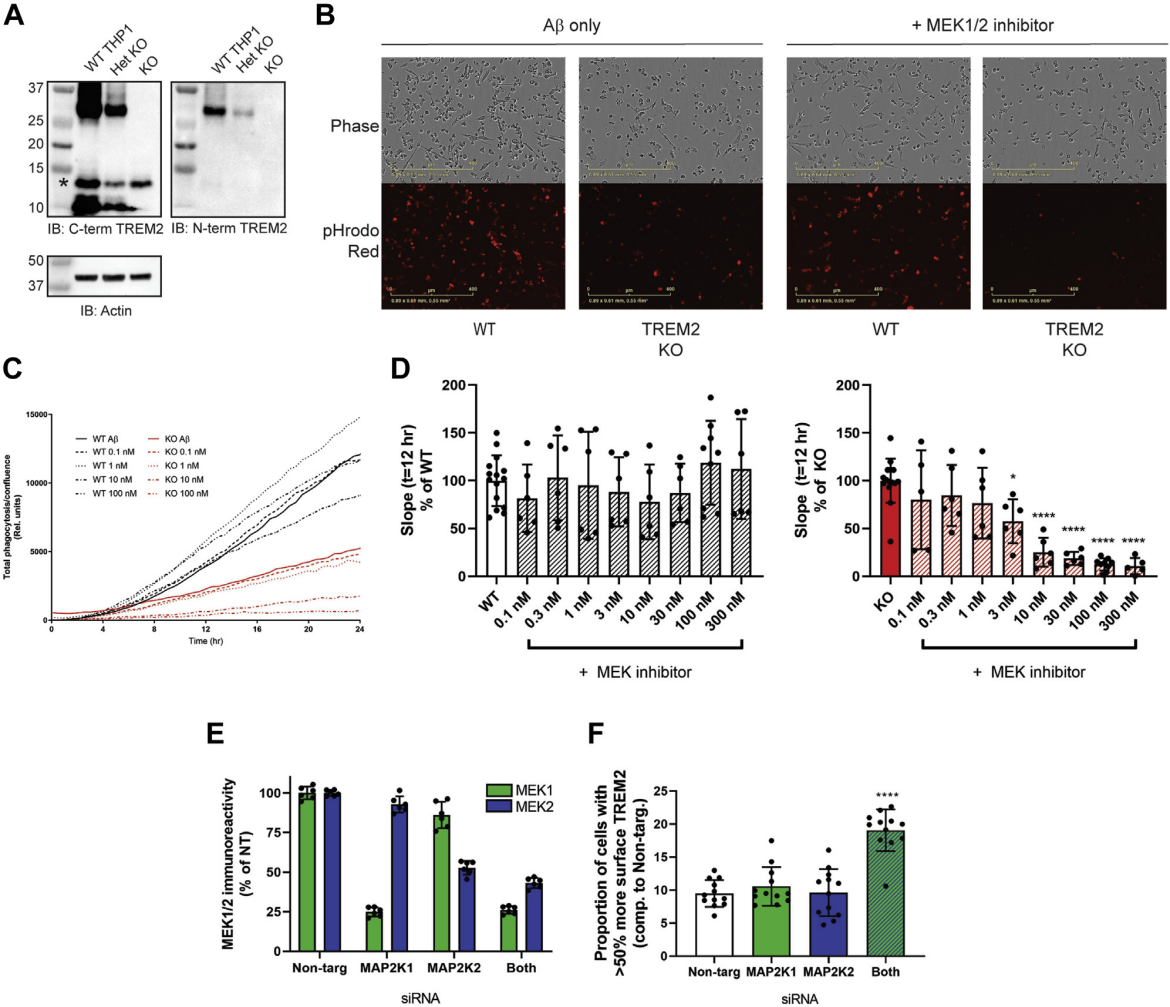
**MEKi-induced TREM2 upregulation at the surface prevents reduced phagocytosis observed in TREM2 KO cells.***A*, western blot analysis of WT, Het KO and TREM2 KO THP1 lines generated by CRISPR/Cas nuclease editing. Both full-length (28 kDa) and a truncated fragment bound by the C-terminal antibody only (∼10 kDa) were reduced and removed in het and TREM2 KO cells, respectively. ∗ - nonspecific band. *B*, images of WT and TREM2 KO THP1 cells following phagocytosis of pHrodo-labeled fibrillar amyloid beta (t = 12 h post-Aβ addition). *C*, representative graph demonstrating total phagocytosis uptake over 24 h, with and without MEK inhibition. *D*, rate of uptake, or slope, in WT cells over the initial 12 h. Values are percentage of untreated cells [n = 9 wells over three experiments, mean ± SD, One-way ANOVA]. *D*, initial rate of uptake in TREM2 KO cells. Values are percentage of untreated TREM2 KO cells [n = 9 wells over three experiments, One-way ANOVA, ∗*p* < 0.05, ∗∗∗∗*p* < 0.0001]. *E*, immunoreactivity for MEK and MEK2 as percent of nontargeting control siRNA (Non-targ), following siRNA treatment for MAP2K1, MAP2K2, or the combination thereof (n = 6 wells). F. Knockdown of MEK1 and MEK2, but not each alone, increased the number of cells with >50% surface TREM2, compared with the mean of nontargeting siRNA treated cells alone (n = 12 wells, mean ± SD, One-way ANOVA, ∗∗∗*p* < 0.0001)

To establish the functional relevance of increased surface TREM2 in myeloid cells, we correlated TREM2 cell surface increase by MEK inhibitors with phagocytosis, a proposed function of TREM2 (18, 19, 20, 21, 22, 23). A phagocytosis assay was developed using pHrodo-labeled fibrillar amyloid beta as a phagocytosis substrate. TREM2 knockout (TREM2KO) cells were generated using CRISPR/Cas editing, and western blot analysis confirmed the absence of TREM2 protein (Fig. 3*A*). In the phagocytosis assay, TREM2 KO cells had noticeably reduced phagocytic capacity over 12 to 24 h compared with WT (Fig. 3, *B*–*C*), agreeing with previous literature (20, 23). Cells were then treated for 24 h with MEK inhibitor to increase surface TREM2 in naïve THP1 cells, prior to adding amyloid beta and evaluating phagocytosis *via* IncuCyte live imaging. The rate of uptake was calculated for the initial 12 h. MEK1/2 inhibition did not significantly change the phagocytosis rate over a range of concentrations that increase surface TREM2 (Fig. 3*D*). However, MEK inhibition dramatically decreased phagocytosis in TREM2 KO cells in a concentration-dependent fashion (Fig. 3*D*). The results show that MEK inhibitors decrease phagocytosis in a TREM2-independent manner, and the lack of effect in the presence of TREM2 suggests a hypothesis in which boosting cell surface TREM2 opposes the TREM2-independent inhibition effects.

Both MEK1 and MEK2 were likely inhibited at the concentrations of MEK inhibitor where a maximal effect on TREM2 was seen (100 nM). To determine if our TREM2 effect was attributable to a specific MEK kinase, siRNA was used to knock down each individually, as well as both together. Naïve THP1 cells were treated with pooled siRNA to each kinase, followed by differentiation, fixation, and cell staining, as performed previously. Staining with MEK1 and MEK2 specific antibodies revealed a significant reduction in kinase expression, with MEK1 being reduced by up to 75% and MEK2 by half (Fig. 3*E*). However, only the combination of MEK1 and MEK2 knockdown could produce a similar effect on surface TREM2 and knocking out each individually had no effect (Fig. 3). Combined with the relative absence of effect of ERK2 inhibitors in our small-molecule screen, the observation that MEK1/MEK2 knockdown is required suggests that a novel target for MEK1/MEK2 may be involved with TREM2 surface recruitment.

**Figure 4:**
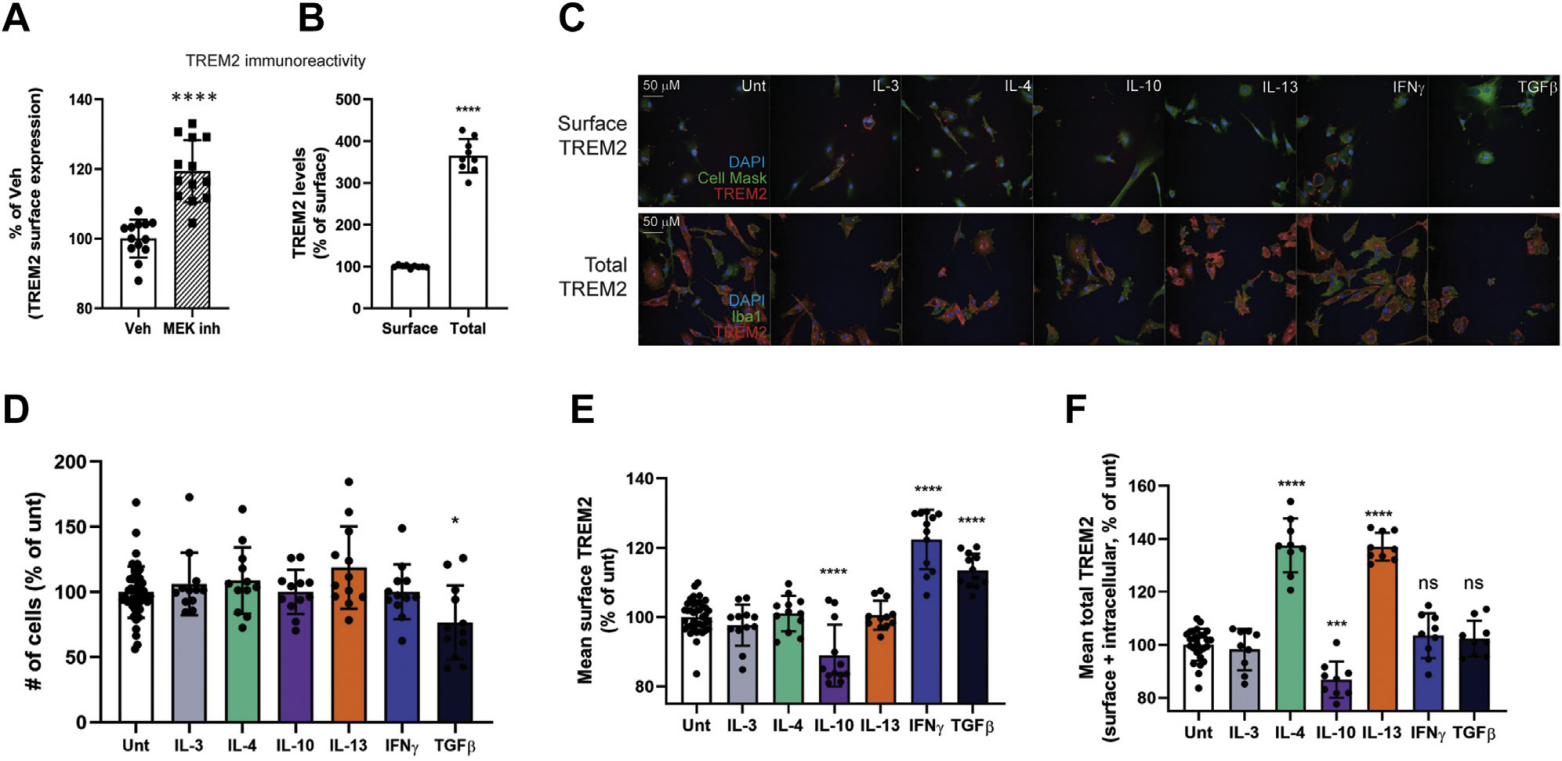
**Cytokine treatment of iMG cells changes TREM2 protein expression and distribution.***A*, compared with THP1 cell line (Fig. 2), iPSC-derived microglia (iMG) cells (14 DIV) have a modest recruitment of surface TREM2 following 100 nM trametinib treatment [expressed as percentage of vehicle, n = 3 experiments, mean + SD, Student *t*-test, ∗∗∗∗*p* < 0.0001]. *B*, intracellular TREM2 is significantly higher than surface TREM2 in iMGs [n = 12 wells from 3 experiments, mean + SD, Student *t*-test, ∗∗∗∗*p* < 0.0001]. *C*, representative images of iMGs treated with various cytokines for 4DIV at day 10 of differentiation prior to immunostaining. *Top panels* show surface TREM2 only (using nonpermeabilizing conditions), *bottom panels* show intracellular + surface TREM2 (staining with 0.2% Triton X-100). 100 ng/ml of each cytokine was used. *D*, number of cells remaining following cytokine treatment, a measurement of cell viability [n = 12 wells from 4 experiments, mean + SD, One-way ANOVA, ∗*p* < 0.05]. *E*, quantification of surface TREM2, expressed as percentage of untreated iMGs [n = 4 experiments, mean + SD, One-way ANOVA, ∗∗∗∗*p* < 0.0001]. *F*, quantification of all TREM2 (intracellular and surface), expressed as percentage of untreated iMGs [n = 12 wells from three experiments, mean + SD, One-way ANOVA, ∗∗∗∗*p* < 0.0001].

To validate results obtained with THP1 cells in a more relevant system, we tested MEK inhibitors in human iPSC-derived microglia-like cells. Protocols to generate microglia-like cells from hiPSCs are commonly used to study microglial biology and represent the most applicable cell-culture-based human microglia model available (for review, see (43)). Microglia-like cells (iMGs) were derived from iPSCs using a previously established protocol (44) prior to testing MEKi-induced surface TREM2 recruitment. Transcriptome analysis revealed a significant upregulation of key microglial genes, which differed substantially from iPSCs differentiated into neurons but was similar to macrophages derived from PBMCs (Fig. S1). iMGs were treated for 24 h with MEK inhibitors and immunostained for cell surface TREM2. Surface TREM2 was raised, but not to the same magnitude as observed in THP1 cells (Fig. 4*A*). This was not due to a lack of intracellular stores of TREM2, as levels within the cell were three to four times greater than at the surface (Fig. 4*B*), which we also observed with THP1 cells (not shown). This suggested that an additional signal may be required to recruit intracellular TREM2 to the cell surface.

Microglia in the brain span a wide range of phenotypes as evidenced by transcriptional profiling, protein expression, and cell morphologies (45, 46, 47, 48). Cytokines are often responsible for changes to microglial phenotypes; therefore, we asked whether iMGs would respond more to MEKi-induced TREM2 recruitment following cytokine treatment to induce a TREM2-requiring phenotype. To test this hypothesis, we treated iMGs with pro- (IFNγ, TGFβ) and anti-inflammatory (IL-3, IL-4, IL-10, and IL-13) cytokines to determine if either cell surface or total TREM2 (surface + intracellular) levels could be modified (Fig. 4*C* for representative images). Only TGFβ appeared to be cytotoxic, as quantified by the average cell numbers remaining per well following treatment (Fig. 4*D*). IL-10 treatment reduced both total and surface TREM2 expression, in agreement with similar observations in osteoclasts (49). Of the rest, IFNγ had the strongest positive effect, significantly increasing surface TREM2 levels (Fig. 4*E*). However, total TREM2 expression was unchanged in IFNγ-treated compared with untreated iMGs, suggesting that the increase in surface TREM2 was due to a redistribution of intracellular TREM2 to the membrane. Interestingly, both IL-4 and IL-13 significantly raised total TREM2 levels but no corresponding increase at the surface was detected (Fig. 4*E*). Therefore, elevating total TREM2 expression alone may not be enough to increase functional TREM2 signaling in iMG cells.

**Figure 5:**
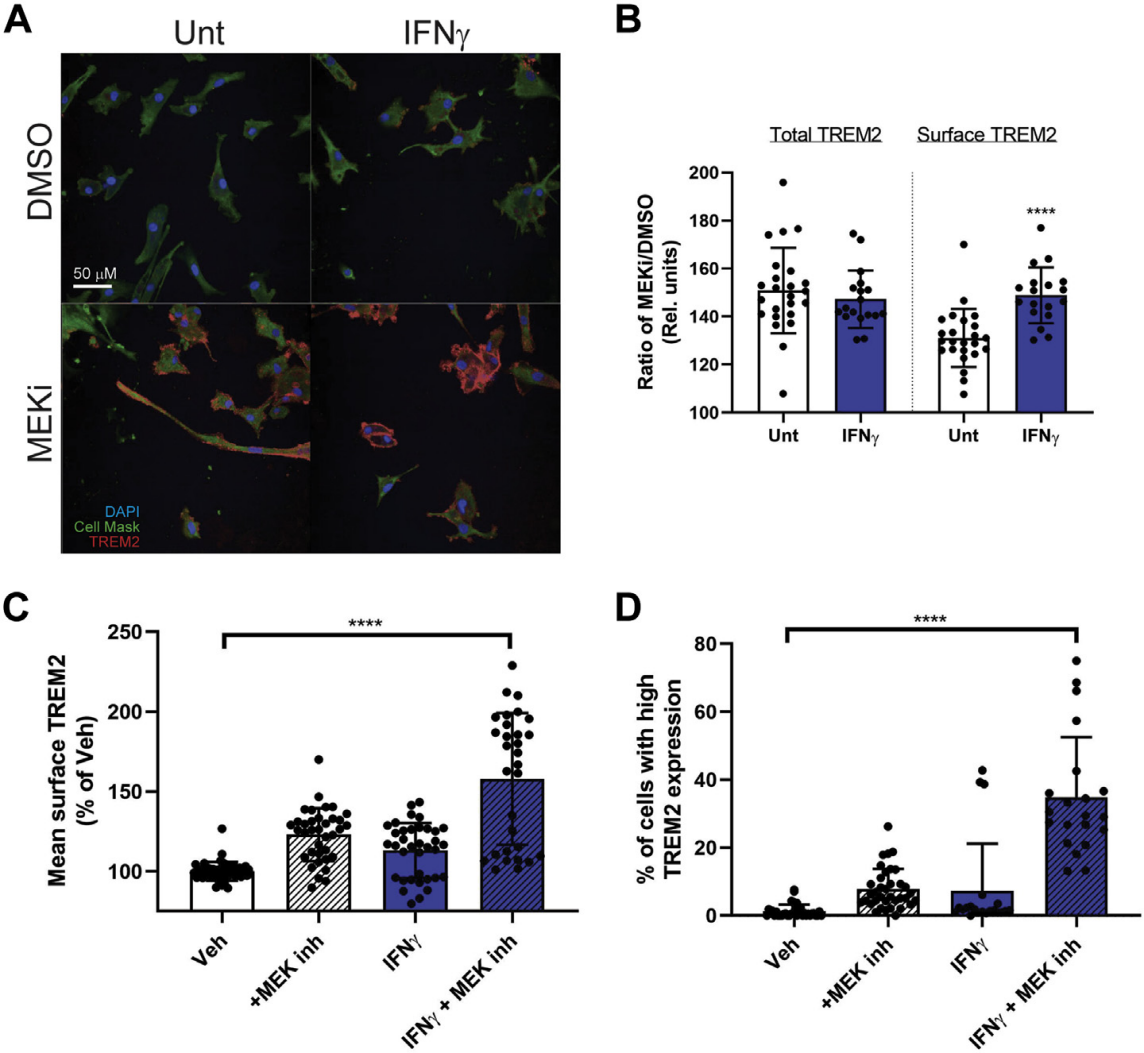
**IFN-primed iMGs are more responsive to MEKi-induced surface TREM2 recruitment.***A*, representative images showing surface TREM2 levels following 4DIV IFNγ treatment and 1 DIV MEK1/2 inhibition. *B*, TREM2 levels as expressed as ratio of MEKi treatment/vehicle treatment, as percentage of vehicle-treated iMGs. [n = 18 wells over 3 experiments, mean + SD, One-way ANOVA, ∗∗∗∗*p* < 0.0001]. *C*, quantification of mean surface TREM2 levels, expressed as percentage of untreated control [n = 30–40 wells from four experiments, mean + SD, One-way ANOVA, ∗∗∗∗*p* < 0.0001]. *D*, quantification of percentage of cells expressing high surface TREM2 levels (>2 SDs over mean of surface TREM2 in vehicle-treated cells) [n = 30–40 wells from four experiments, mean + SD, One-way ANOVA, ∗∗∗∗*p* < 0.0001].

We were interested to see if IFNγ-primed iMGs would also respond to MEKi treatment, boosting surface TREM2 levels beyond that induced by IFNγ. Other time points and concentrations of IFNγ were tested, and 4 DIV and 100 ng/ml were considered optimal (not shown). Following a 4DIV treatment of IFNγ, iMGs were treated with MEKi for an additional 24 h prior to immunocytochemistry (Fig. 5*A*). An analysis of surface *versus* total TREM2 following MEKi revealed that MEK inhibition in IFNγ-primed iMGs could further increase surface TREM2 but did not change total TREM2 expression (Fig. 5*B*). MEKi increased levels more in cells primed with IFNγ (Fig. 5*C*), significantly increasing surface TREM2 compared with untreated iMG cells. The heterogeneity of myeloid cells resulted in high variability of surface TREM2 expression, and so the proportion of cells that showed elevated TREM2 cell surface expression, defined as more than 2 standard deviations above the average in vehicle-treated wells, was measured. This analysis revealed that a greater percentage of cells in culture expressed elevated TREM2 (Fig. 5*D*). Thus, IFNγ and MEKi treatment had an additive effect on cell surface TREM2 expression.

## Discussion

Strategies to target microglia biology, including TREM2, in AD have intensified over recent years (50). Novel approaches to activate TREM2 signaling could have a significant impact on future drug development. In this study we show that it is possible to double cell surface TREM2 protein on human myeloid cells, the largest magnitude of increase in cell surface TREM2 that has been described. There is a wealth of knowledge from TREM2-deficient mice, but far less is known about human myeloid cell responses to TREM2 perturbations, and thus we chose to limit our work to human cells. GWAS studies show that TREM2 can be a significant risk factor for AD (1, 2), and its ability to promote several vital microglia functions indicates that elevating TREM2 activity might promote microglial homeostasis (18). Our results reveal a cell signaling pathway that can regulate TREM2 trafficking to the cell surface, specifically through the kinases MEK1 and MEK2, and the extended time point used here (24 h) implies a stable, nontransient effect on surface TREM2 expression. It is unclear what contribution TREM2 cleavage plays in our *in vitro* model systems, and levels may be increased even further if TREM2 cleavage was reduced. In addition, given the well-documented role of TREM2 in phagocytosis (19, 20, 21, 22, 23), the observed prevention of depressed phagocytosis caused by canonical MEK inhibition in WT myeloid cells, which could not be replicated in TREM2 KO cells (Fig. 3), suggests that this enhanced TREM2 population is functionally relevant. These data support the hypothesis that increasing TREM2 membrane expression may alter microglia phenotypes with therapeutically beneficial consequences.

MEK1/2 signaling is well understood, and inhibitors of the pathway, and of MEK1/2 specifically, are used as cancer therapeutics (for review, see (41)). Some may derive significant potency and specificity as a result of binding to an allosteric site, with IC_50_ values in the low nM range. The IC_50_ values that we observed for increasing surface TREM2 expression were also low nM, and a number of structurally diverse compounds had similar activity, suggesting that the primary if not exclusive mechanism of the compounds was MEK inhibition. ERK-specific inhibitors did not alter TREM2 expression at relevant concentrations, (Fig. 2*F*), and no other substates for MEK1 or MEK2 besides ERK1/2 have been reported (42), which hints at a novel MEK1/2 signaling mechanism that modulates TREM2 independently of ERK. This is supported by the observation that, despite the similarity in structure and function between the two MEK kinases, MEK2 KO animals develop normally, whereas MEK1 KOs do not (51), further suggesting a divergence in function. The suppressive role of canonical MEK/ERK signaling on phagocytosis in myeloid cells has been reported (52) and may align with the TREM2-independent inhibition that we observed. The ERK-independent link between MEK kinases and TREM2 observed here merits further investigation.

Multiple studies have demonstrated that TREM2 KO reduces phagocytosis, suggesting that increasing TREM2 surface expression might enhance the process. Indeed, increased gene dosage elevates phagocytosis in BAC-TREM2 mice (37). Comparing wild-type and TREM2KO cells suggests this relationship but the lack of a strong effect upon increasing surface TREM2 with MEK inhibitors is puzzling. This might be explained by mechanisms related specifically to MEK1/2 signaling. Activation of TREM2 increases ERK phosphorylation (53), and thus MEK inhibitors might simultaneously elevate membrane TREM2 expression and inhibit subsequent TREM2 pathway activation. Consistent with this, a recent CRISPR screen designed to discover phagocytosis regulators revealed that KO of MAPK1, which encodes ERK2, drastically reduces phagocytosis of multiple substrates (54). Maintaining ERK signaling while simultaneously increasing TREM2, by knockdown of either MEK1 or MEK2 individually, did not increase TREM2 expression (Fig. 3*F*). Therefore, stimulation of phagocytosis by MEK inhibition may not occur despite stimulation of surface TREM2 expression. But it does suggest a biological rationale for TREM2 recruitment in microglia cells. Depression of phagocytosis achieved through inhibition of ERK signaling may result in a reflex recruitment of surface TREM2 that counteracts the reduction. Whether this is specific to ERK signaling or could be a consequence of inhibition of phagocytosis by other mechanisms needs to be investigated.

Microglia are dynamic cells that readily alter their phenotype in response to their external environment. The modest initial response to MEK inhibition in naïve iMG cultures suggests that these *in vitro* microglia as used lack the mechanisms that mediate TREM2 responsiveness to MEK inhibitors. But the large intracellular protein stores compared with surface expression (Fig. 5*A*) suggest that the cell is ready if properly primed. Interferon signaling is often associated with microglial priming, a preparatory signaling event that alters several myeloid processes including surface receptor recruitment (55). Both type I and type II interferon microglia signatures have been detected in neurodegeneration (56), and blocking interferon signaling can prevent the development of neuroinflammation and DAM formation (57). Thus, microglia priming with IFNγ may be an early event in AD progression resulting in increased DAM-associated transcripts such as TREM2 (10, 58), and IFNγ-stimulated microglia may be more responsive to MEKi-induced TREM2 recruitment. A worsening of AD pathology in the absence of TREM2 implies this transition is a necessary biological event that could be assisted by therapeutic intervention (10). Whether our observations are only a feature or limitation of our cell culture system will require future study, perhaps addressed by examining the responsiveness to TREM2 recruitment in *ex vivo* microglia of various phenotypes. If this phenomenon holds true, designing TREM2-specific drugs may be aided by the notion that only disease-relevant microglia, such as those primed by interferon signaling and need to be targeted, will respond. But it may also complicate treatment, as the therapeutic window could shift considerably as the brain microglia profile shifts with disease progression.

TREM2 mutations that elevate AD risk seem to be partial loss of function, suggesting that boosting TREM2 function may be efficacious in the disease. If so, the degree of such enhancement that would be required for significant efficacy is unknown. A total loss of TREM2 function results in NHD. In contrast, carriers of heterozygous AD-associated mutations still have one normal functioning copy of the gene, and early disease onset is not observed as in those with familial PS1 or APP mutations (59, 60). Further, the mutant copy of the gene may produce protein of at least partial function, yet still confer significant disease risk. Immunotherapy designed to prevent TREM2 cleavage only increased surface TREM2 expression by ∼25 to 50%, but this was sufficient to significantly increase microglial plaque coverage in APP-NL-G-F mice (39). Considering this, we speculate that a modest increase in TREM2 activity, if it could be achieved therapeutically, may have a significant benefit, although at what stage in the progression of the disease such a therapeutic benefit might be realized is a matter of conjecture. One caveat to bear in mind is that TREM2 mutants are rare, and the overwhelming majority of AD patients carry two copies of the common variant (61). It is not known whether boosting TREM2 above background levels would be as beneficial as restoring function in a mutated TREM2 situation. Still, considering that heterozygous mutations show intermediate effects, and the possibility that common variant carriers have diminished TREM2 function by unknown mechanisms, it is reasonable to speculate that increased TREM2 activity above baseline may be beneficial, and that the approximately twofold increase in cell surface TREM2 that we observed with MEK inhibition may be significant. Supporting this supposition will require finding specific TREM2 modulators and mechanisms that do not otherwise significantly impact normal microglial function.

## Experimental procedures

### Cell culture

THP1 cultures were purchased from ATCC. TREM2 KO THP1 cells were generated by CRISPR nuclease-induced targeted double-strand break at the Genome Engineering and iPSC center (Washington University in St Louis). Exon 2 of TREM2 was targeted using guide RNA 5’-GAAGTCTGCCCACGGGTTTTNGG-3’. Sequencing determined that all three alleles were disrupted, with two alleles having a 1 bp insertion, and the third having a 1 bp deletion. No off-targets were detected in clones with 2 bp mismatch or fewer. For all experiments, only lines with all three TREM2 alleles disrupted were used. Cells were maintained in RPMI media supplemented as described on ATCC website. For experiments with differentiated cells, THP1 cells were plated at 30k/well in 96-well Perkin Elmer Cell Carrier imaging plates in 100 nM PMA for 24 h. Media was completely replaced without PMA and rested for an additional 24 h prior to starting experiments. Multiple MEK inhibitor compounds were used throughout this study, including those listed in Figure 2, binimetinib for Figure 3, and trametinib for Figures 4 and 5.

mRNA knockdown was performed using Dharmacon Accell SMARTPool siRNA delivery per manufacturer’s instructions for suspension cells, with two modifications: 1) siRNA was applied at 5 μM instead of 1 μM, and 2) in the cells’ normal serum-containing media. Cells were treated with siRNA for a total of 4 days prior to PMA differentiation, then media changed 1 day later. After an additional 24-h rest period, cells were fixed for immunocytochemistry.

### Small molecule annotated library screen

Differentiated THP1 cells were treated with compounds by diluting the 29K compound annotated library in fresh complete media and adding volume to each well using a Beckman BioMek FX liquid handler for a final compound concentration of 10 μM. Following a 24 h incubation, cells were fixed in 2% PFA, washed in PBS, and probed with a TREM2 commercial antibody (Cat#AF1828, R&D Systems) and CD33 commercial antibody (Cat#366602, BioLegend) overnight with washes and Alexa Fluor-647 conjugated secondary antibody incubation the next day. Cells were imaged at 20× with a Cellomics Arrayscan VTI and immunoreactivity for TREM2 and CD33 was quantitated using mean average intensity TREM2 and mean ring spot total area for CD33. Toxicity was measured using valid nuclear count with 5× objective. Hits of interest were validated as previously described with a 6-point titration.

### Surface biotinylation

Differentiated THP1 cells were treated with MEK inhibitor for 24 h, followed by washing with PBS and treating with sulfo-NHS-SS-biotin (2.5 mg/ml, ThermoFisher) for 30 min at 4 degrees. Cells were quenched and washed in cold 100 mM glycine. Cells were lysed and incubated with Neutravidin (ThermoFisher) overnight on nutator. Beads were washed, eluted with Laemmli buffer, and immunoblotted with TREM2 antibody (cat #91068, D814C, Cell signaling).

### iPSC-derived microglia (iMGs)

iMGs were generated as described (44). Briefly, iPSC cells were converted into embryoid bodies (EBs) by seeding them in AggreWell 800 cell culture plates (StemCell Tehnologies) using XVivo media supplemented with 50 ng/ml BMP4, 20 ng/ml SCF, and 50 ng/ml VEGF growth factors. A half-media change was performed every day for 5 days, at which point EBs were dislodged and seeded into 150 cm^2^ flasks. EBs were maintained in these flasks for 3 to 4 weeks before myeloid precursor cells were generated and secreted into the media. At this point, cell-containing media was transferred to multi-well imaging plates and differentiated into microglia cells with 10 ng/ml GM-CSF and 100 ng/ml IL-34. Microglia-like cells were used after 2 weeks of differentiation, performing half-media changes every 3 to 4 days.

### Phagocytosis assay

Recombinant human Aβ42, labeled with pHrodo Red (ThermoFisher), was synthesized by New England Peptide Inc (Gardner, MA). Single peptide labeling was achieved by modification of the amino acid sequence (S8C), and purity was verified by spectral analysis at the vendor. The labeled peptide was provided as a lyophilized monomers and was aggregated to a fibrillar format using resuspension in DMSO, followed by 2 days incubation at 37 °C in 10 mM HCl, 150 mM NaCl. Fibrillar peptide format was assessed using electron microscopy as compared with monomeric suspensions. Differentiated THP1 cells were treated with MEK1/2 inhibitors at 100 nM for 24 h prior to media change with fresh inhibitor and 500 ng/ml pHrodo-labeled Aβ. Images were taken every 30 min in IncuCyte live-imaging platform (Essen).

### Immunocytochemistry

Following treatment, cells were fixed in 2% PFA/4% sucrose in PBS for 30 min with rocking. Cells were washed twice with PBS and blocked for 1 h in 5% donkey serum in PBS. Antibodies to TREM2 (1:200; #AF1828, R&D) and CD33 (1:200; #366602, BioLegend) were added overnight. Plates were washed with PBS four times before incubating with secondary antibodies (1:500), Cell Mask (1:2000), and Hoescht nuclear stain (1:2000) for 1 h. Following four additional washes, cells were imaged on Opera Phenix (Perkin Elmer) or Cellomics (Thermo Fisher) high-content imagers. To assess intracellular TREM2 or MEK1/2 levels, Triton X-100 (0.2%) was added to blocking buffer and wash steps.

## Data availability

All data are contained within the article and supporting information.

## Conflict of interest

The authors declare that they have no conflict of interests with the contents of this article. All authors are employees of AbbVie. The design, study conduct, and financial support for this research were provided by AbbVie. AbbVie participated in the interpretation of data, review, and approval of the publication.
