# Supplementary material for: MEK1/2 activity modulates TREM2 cell surface recruitment
Source: J Biol Chem. 2020 Dec 25;296:100218. doi: 10.1074/jbc.RA120.014352 (PMC7948395; doi:10.1074/jbc.RA120.014352)
Supplement: Figure S1 [file mmc1.pdf]

TREM2 cell surface recruitment is modulated by MEK1/2 kinase signaling

Jason Schapansky[1*], Yelena Y. Grinberg[1], David M. Osiecki[2], Emily A. Freeman[1], Stephen G. Walker[2], Eric Karran[1], Sujatha M. Gopalakrishnan[2], Robert V. Talanian[1]

Supporting information – Supplemental figure 1 and legend

Supplemental Figure 1

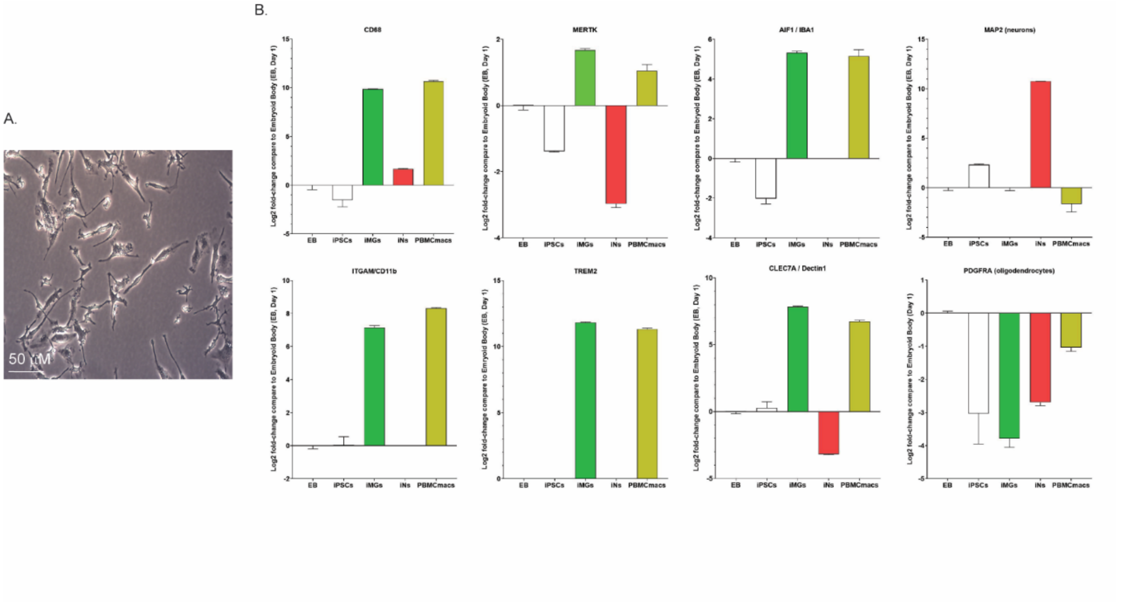

Supplemental figure 1. Characterization of iMG cultures. A. Representative image of induced pluripotent stem cell-derived microglia (iMG) culture. Cells had ramified-like morphology indicative of microglia cells. B. Select qPCR analysis of select microglia and non-microglia genes. Comparisons were made between the following cell types: iPSC, induced pluripotent stem cell; iN, iPSC-derived neurons; PBMCmacs, peripheral blood monocytic cells differentiated into macrophages. Values were normalized to embryoid bodies (EBs), the structure formed from iPSCs as the first step in the iMG differentiation protocol.
